# Influence of hormonal treatments on progesterone levels to enhance embryo survival and kidding rates in goats

**DOI:** 10.5713/ab.24.0578

**Published:** 2024-11-06

**Authors:** Manita Wittayarat, Navapol Kupthammasan, Hakim Jehdo, Ratree Jintana, Sopita Suttikrai, Niraporn Tongkumtae, Nantarat Chutijiratthitkan, Pokchon Khirilak, Sirirat Norsoongnern, Supitcha Kaewma, Chaiyawan Wattanachant, Saritvich Panyaboriban

**Affiliations:** 1 Faculty of Veterinary Science, Prince of Songkla University, Songkhla, Thailand; 2 Small Ruminant Research and Development Center, Faculty of Natural Resources, Prince of Songkla University, Songkhla, Thailand; 3 Research and Development Center for Livestock Production Technology, Animal Hospital and Student Training Center, Faculty of Veterinary Science, Chulalongkorn University, Nakhon Pathom, Thailand; 4 Songkhla Artificial Insemination and Biotechnology Research Center, Department of Livestock Development, Songkhla, Thailand; 5 Animal Production Innovation and Management Division, Faculty of Natural Resources, Prince of Songkla University, Songkhla, Thailand; 6 Department of Anatomy, Faculty of Veterinary Science, Chulalongkorn University, Bangkok, Thailand

**Keywords:** Embryo Transfer, Fertility, Goat, Hormonal Treatments, Progesterone, Reproductive Outcomes

## Abstract

**Objective:**

This study investigated the efficiency of different estrous synchronization programs and hormonal treatments in improving reproductive outcomes in goats. Conducted on a commercial farm in southern Thailand, the study used multiparous Shami and Anglo-Nubian breed goats.

**Methods:**

In experiment 1, goats were randomly allocated to two estrous synchronization treatments: 11-day (CI11D) and 13-day (CI13D) intravaginal progesterone implants, followed by artificial insemination (AI) with frozen-thawed semen. Various hormonal treatments (human chorionic gonadotropin [hCG], gonadotropin-releasing hormone [GnRH] analogue, progestogen) were administered on day 5 post-AI to elevate progesterone levels. Experiment 2 assessed embryo survival after transferring early- and late-stage embryos, using GnRH analogue to increase progesterone levels in the recipient goats.

**Results:**

Results showed that GnRH analogue significantly increased pregnancy rates, kidding rates, and the number of kids born in the CI13D group. Progesterone levels were higher in treated groups, particularly with GnRH analogue, though estradiol levels varied between synchronization protocols. Late-stage embryo transfers significantly improved pregnancy rates and reduced embryonic loss compared to early-stage transfers. GnRH analogue supplementation reduced early embryonic mortality, enhancing embryo survival and overall reproductive performance.

**Conclusion:**

This study demonstrates the efficacy of tailored estrous synchronization and hormonal treatments in optimizing goat reproductive outcomes, with significant implications for improving fertility management in commercial goat farming. Although no significant differences were observed in progesterone levels, the administration of GnRH analogue reduced early embryonic mortality and improved reproductive outcomes, demonstrating its potential to enhance embryo survival and reproductive performance in goats.

## INTRODUCTION

The dairy and meat goat industries worldwide are experiencing rapid growth. By 2013, the global goat population had surpassed one billion, reflecting a 34% increase since 2000. This growth is driven by changing human incomes, evolving food preferences, and the impact of climate change, which has restricted areas suitable for cattle farming [1]. Thailand has an estimated total goat population of around 440,000 with the majority located in central Thailand, near Bangkok, and in the southern region of the country [2]. The goat industry in Thailand is predicted to expand concurrently with the nation's economy in the next few decades. The use of artificial insemination (AI) and embryo transfer techniques has become essential for maximizing assisted reproductive outcomes and promoting the growth of the small ruminant industry [3,4]. However, there are several areas where protocols need improvement, such as estrus control and synchronization, as well as addressing insufficient progesterone production during pregnancy.

Progesterone and its analogues play a crucial role in controlling the reproductive systems of various animal species, serving to synchronize births, inducing fertile estrus, and facilitating the use of assisted reproductive techniques [5]. While several studies have demonstrated a highly estrous response and increased pregnancy rates in goats with the use of progesterone-impregnated controlled internal drug-releasing (CIDR) devices for estrous induction, there is notable variability in the duration of treatment, ranging from 5 to 16 days [5]. Without gonadotropin treatment, Nubian goats treated with CIDR for 13 days showed a 100% estrus rate [6]. In comparison, Saanen goats treated with CIDR for 11 days exhibited an 83% estrus response and a 74% pregnancy rate [7]. This variability highlights the need for further research to optimize CIDR protocols for goats and potentially enhance reproductive outcomes in this species.

Inadequate progesterone production during pregnancy, particularly prominent in the non-breeding season [8], is another significant limitation in goat breeding. This deficiency contributes to 30% to 40% of embryonic deaths and decreased pregnancy rates in small ruminants [9–11]. Various therapeutic approaches have been employed to elevate progesterone levels and enhance luteal function [12]. Improved luteal function resulting from progestogen treatment has a positive impact on both the fertilization process and the conception rate in goats [13]. Administering luteotropic hormones like gonadotropin-releasing hormone (GnRH) during both the early [14–16] and late luteal phases [17,18] triggers the release of luteinizing hormone (LH) and follicle-stimulating hormone (FSH). This leads to elevated serum progesterone levels and the formation of an accessory corpus luteum (CL) in sheep ovaries [12]. While increased progesterone levels are recognized to enhance embryonic survival and reduce losses in ruminants, the outcomes of hormonal treatments in various experimental settings are often inconsistent, conflicting, and inconclusive [7]. These variations highlight a critical need for further research to better understand and optimize these therapeutic interventions. This study addressed these inconsistencies by evaluating different hormonal protocols to improve conception rates and reduce pregnancy loss in goats. We examined whether increasing progesterone levels using hormones such as human chorionic gonadotropin (hCG), GnRH analogues, or progestogen could produce more consistent results in embryo survival and offspring production. Our study offers practical solutions for improving reproductive efficiency in commercial goat production.

## MATERIALS AND METHODS

### Location, and animals

This study was carried out on a commercial goat farm, located in southern Thailand, at latitude 7° 11' 55.75" north, longitude 100° 35' 42.36" east. All animal procedures were approved by the Institutional Animal Care and Use Committee (IACUC at the Prince of Songkla University (MHESI 68014/1339). All animals were multiparous Anglo-Nubian breed goats, ages ranging from 1 to 4 years, with at least 60 days after previous parturition. They also had body condition scores ranging from 2.5 to 3.5 on a scale of 1 to 5.

### Experimental design

**Figure 1:**
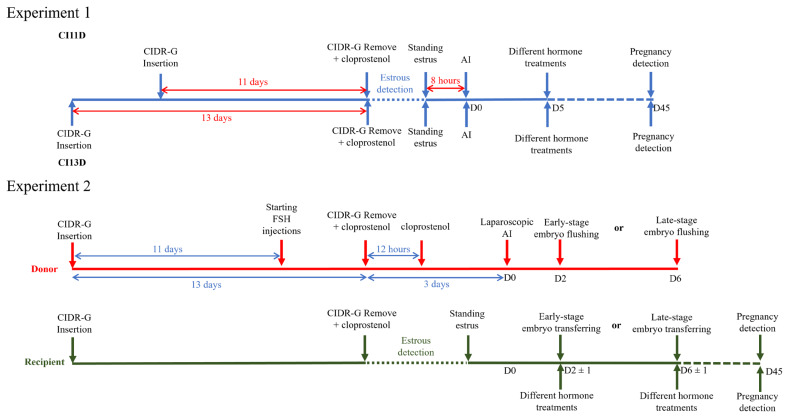
Schematic overview of the experimental designs for Experiment 1 and Experiment 2 in this study. In Experiment 1, different hormone treatments were administered to four groups: a control group (1 mL of 0.9% sterile normal saline, IM), an hCG group (300 IU of hCG), a GnRH analogue group (0.05 mg of gonadorelin), and a progestogen group (receiving CIDR-G [ intravaginal insertion for 15 days; D5 to D20). In Experiment 2, the hormone treatments were divided into two groups: a control group and a GnRH analogue group. CI11D, inserting the CIDR for 11 days; CIDR, controlled internal drug-releasing; CI13D, inserting the CIDR for 11 days; IM, intramuscularly; hCG, human chorionic gonadotropin; GnRH, gonadotropin-releasing hormone.

Experiment 1 was designed to compare the hormonal changes and reproductive outcomes (pregnancy rate, kidding rate, and number of kids) in doe goats that were increased progesterone levels with various hormones (hCG, GnRH analogue, and progestogen) during two different estrous synchronization programs and AI with frozen-thawed semen. Experiment 2 was to assess the effectiveness of this program in reducing embryonic losses at both early and late embryo stages in recipient goats after embryo transfer. Figure 1 illustrates the experimental designs for Experiment 1 and Experiment 2 conducted in this study.

### Estrous synchronization programs

In experiment 1, goats (n = 64) were randomly allocated into two different estrous synchronization treatments, with each group receiving an intravaginal progesterone implant inserted for either 11 days (CI11D) or 13 days (CI13D). Briefly, the goats were treated with intravaginal progesterone implants (CIDR-G, Eazi-Breed, containing 0.3 g progesterone in silicone; InterAG, Hamilton, New Zealand) for either 11 days or 13 days. Upon removal of the CIDR-G, each goat received an injection of 30 μg cloprostenol (Estrumate; Schering-Plough Animal Health, Kenilworth, NJ, USA). Standing estrus was detected by a teaser buck twice daily (morning and evening). Intracervical AI with frozen-thawed semen was conducted 8 hours later (day 0: D0). On day 5 (D5), each doe in both the CI11D and CI13D groups was randomly assigned to one of four groups: a control group (1 mL of 0.9% sterile normal saline intramuscularly [IM]), an hCG group (300 IU of hCG [Chorulon; Intervet, Boxmeer, Netherlands] IM), a GnRH analogue group (0.05 mg of gonadorelin (Fertagyl; MSD Animal Health, Rahway, NJ, USA) IM), and a progestogen group (receiving CIDR-G intravaginal insertion for additional 15 days).

### Semen preparation and intracervical artificial insemination

Semen was collected from healthy fertile goat bucks (Shami and Anglo-Nubian breeds). The protocol for semen collection and evaluation followed the procedure outlined by Kupthammasan et al [19]. Semen freezing and thawing procedures were conducted according to Anakkul et al [20], with minor modifications. The semen dose was prepared to contain 100 million motile spermatozoa.

At the time of insemination, the vulvar region of each estrous doe was cleaned, and then a vaginal speculum with lubrication was inserted through the vagina to visualize the cervical opening under a light source. Subsequently, frozen-thawed semen was inseminated, passing through the cervical opening and deposited from the mid-cervix into the body of the uterus.

### Blood sampling, plasma progesterone and estradiol 17-β assays

In experiment 1, starting from the date of CIDR-G insertion to 20 days after AI, blood was collected from the jugular vein of all animals in each group at nine time points and kept in heparinized tubes. Plasma was immediately separated by centrifugation at 1,500×g for 15 min and stored at −20°C until analyzed. Plasma progesterone and estradiol 17-β were determined using the radioimmunoassay (RIA) procedure previously described by Suthikrai et al [21]. The reliability of progesterone and estradiol 17-β determination were tested in three pools of low, medium, and high standard progesterone or estradiol 17-β added to the blank plasma pools. The specificity of the progesterone antibody (#AS-P 15P/040; Dr. Hilary Dobson, Cheshire, UK) used for analysis was defined by the cross-reactivity data obtained in the described RIA system, as follows: progesterone 17-α, 100%; hydroxyprogesterone, 0.74%; and corticosterone, 0.68%. The coefficients of variation of the three pools of internal control of the progesterone assay were 9.92%, 6.38%, and 4.78%, while the inter-assay coefficients of variation of the progesterone assay were 13.00%, 11.03%, and 7.39%. The sensitivity of the progesterone assay was 0.05 ng/ml. The intra- and inter-assay coefficients of variation were 4.83 and 8.40% for estradiol 17-β, respectively. The specificity of estradiol 17-β antibody (#8932 180381; Dr. R.I. Cox, CSIRO Division of Animal Production, Prospect, NSW, Australia), defined as the cross-reactivity data obtained in the described RIA system, was as follows: Oestradiol 17-β, 100%; Estradiol 17-α, 0.48%; Estrone, 8.40%; and other steroids less than 1.68%. The sensitivity of the estradiol 17-β assay was 1.46 pg/mL.

### Superovulation and embryo production protocols

All donors were superovulated using a technique based on Panyaboriban et al [22] with minor modifications. Briefly, a CIDR-G device was inserted intravaginally for 13 days. FSH (Folltropin-V; Bioniche Animal Health, Belleville, ON, Canada) was divided into six doses (50, 50, 30, 30, 10, and 10 mg), and the first dose was administered on day 11 of CIDR-G insertion. Subsequently, two doses of 125 μg cloprostenol (Estrumate; Merck Animal Health, Rahway, NJ, USA) were administered IM: one at the time of CIDR-G removal and the other 12 hours later. Three days after CIDR-G removal, laparoscopic AI was performed on all donors using a direct-view 5 mm laparoscope (Karl Storz Veterinary Endoscopy, Tuttlingen, Germany). The procedure followed the anesthetic and AI protocol described by Panyaboriban et al [22].

Early-stage embryos were collected on day 2 after insemination by direct oviduct flushing, while late-stage embryos were collected on day 6 after insemination by uterine flushing. The flushing media was prepared with Dubecco’ phosphate-buffered saline (Gibco, Grand Island, NY, USA) supplemented with 2% (v/v) fetal bovine serum (JR Scientific, Inc., Woodland, USA). The embryo-collecting procedures were those described by Panyaboriban et al [22] and Khunmanee et al [23]. After collection, the embryos were immediately searched under a stereomicroscope (Nikon SMZ745; Nikon, Tokyo, Japan) with 10X magnification. The recovered embryos were evaluated and graded according to agreed the International Embryo Technology Society conventions. All recipients were estrus-synchronized using the same protocol as mentioned above.

### Embryo transfer protocol

Two good quality embryos were selected and freshly transferred to one recipient. Early-stage embryos were transferred into the oviduct, while late-stage embryos were transferred into the cranial one-third area of the uterine horn on the side where the corpus luteum had appeared. Embryo transfers were performed only on recipients that were in estrus within ±1 day compared to the donors, with early-stage embryos transferred on day 2 post-estrus and late-stage embryos transferred on day 6 post-estrus.

### Pregnancy detection

Forty-five days after insemination or transfer, the does were pregnancy diagnosed by real-time B-mode ultrasonography using a transcutaneous probe. In pregnant does, fetal bodies or placental landmarks (placentome maturation into a C-shape or a hollow circle) were observed within enlarged, fluid-filled uterine sections [22].

### Statistical analysis

All statistical analyses were performed using the R programming language (version 4.1.2) and RStudio (version 2022.07. 0+548). The normal distribution of data was checked using the Shapiro-Wilk test. In experiment 1, a randomized complete block design was used. The quantitative data of treatments, including pregnancy rates, kidding rates, and number of kids, were analyzed using two-way analysis of variance (ANOVA), accounting for the factorial arrangement (2×4) of the treatments. For post hoc comparisons, the Tukey-Kramer test was applied, or the Wilcoxon signed-rank test was used when there was unequal variation among treatments. Plasma hormone levels (progesterone and estradiol 17-β) were analyzed by two-way repeated measures ANOVA. In experiment 2, pregnancy, kidding, and embryonic loss rates were tested for statistical significance using the Chi-square test (χ^2^). Differences between experimental groups were considered statistically significant when p<0.05.

## RESULTS

### Comparison of various hormones with reproductive outcomes after insemination

The estrous synchronization program used both in CI11D and CI13D was found to be highly effective. All goats that were subjected to the program exhibited signs of estrus within 48 hours after the CIDR-G removal. The estrus rate for the CI13D group was 100% (32/32), while the estrus rate for the CI11D group was 94.12% (32/34). The average onset of estrus in CI11D and CI13D groups was 33.56±10.16 hours and 31.57±10.73 hours, respectively. The pregnancy rate, kidding rate, number of kids, and number of kids per litter are shown in Table 1. In the CI13D, the result found that the use of GnRH analogue hormone significantly increased the pregnancy rate, kidding rate, and the number of kids born compared to the control group (p<0.05). Additionally, it yielded better results than the hCG and progesterone groups (p>0.05). As for CI11D, it was found that the GnRH analogue group also yielded the best results, although the differences were not statistically significant. When comparing the CI11D and CI13D programs using each type of hormone, it was found that each pair showed a tendency for tend to better results in the CI13D group in terms of pregnancy rate and the total number of kids born. This was particularly evident in the group using GnRH analogue hormone in day 5 after insemination.

### Comparison of various hormones with hormone levels

**Figure 2:**
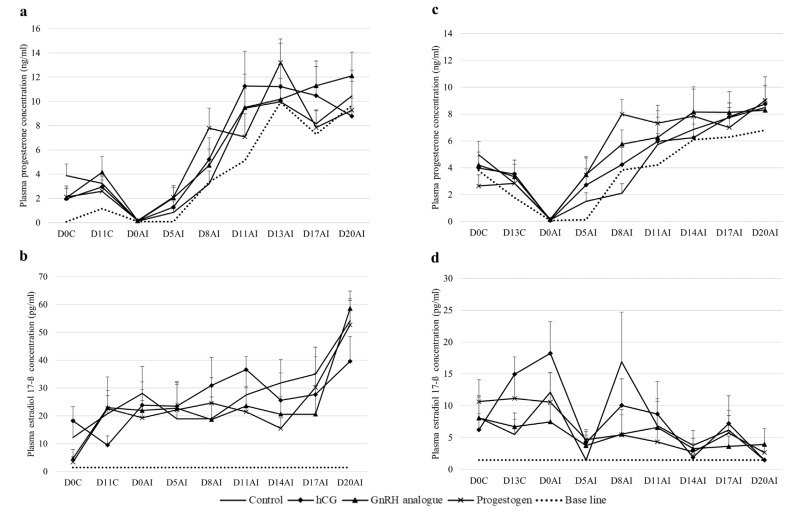
Mean plasma concentrations of progesterone and estradiol 17-β throughout the estrous synchronization protocol in goats, comparing levels to baseline across four groups: control, hCG, GnRH analogue, and progestogen. Progesterone levels are shown for the CI11D program (a) and the CI13D program (c), while estradiol 17-β levels are depicted for the CI11D program (b) and the CI13D program (d). D0C, day 0 at CIDR insertion; D11C, day 11 of CIDR insertion; D0AI, day 0 of AI; D5AI, day 5 after AI; D8AI, day 8 after AI; D11AI, day 11 after AI; D13AI, day 13 after AI; D17AI, day 17 after AI; D20AI, day 20 after AI; hCG, human chorionic gonadotropin; GnRH, gonadotropin-releasing hormone; CI11D, inserting the CIDR for 11 days; CIDR, controlled internal drug-releasing; CI13D, inserting the CIDR for 11 days.

Figure 2 illustrates the mean plasma concentrations of progesterone and estradiol 17-β hormones throughout the days of the estrous synchronization protocol across different treatment groups in goats both in the CI11D and CI13D programs. Progesterone levels in all groups declined to baseline on the insemination date and then continually increased over the period following insemination (F-value = 20.77, p<0.01). However, all treatment groups tend to have higher progesterone levels than the control group, especially in the GnRH analogue group. For estradiol, it was found that in the CI13D, estradiol levels declined to baseline on days 5 and 14 after insemination in all groups. However, in the CI11D, there was no decline in estradiol levels throughout the hormone monitoring period.

### Effect of gonadotropin-releasing hormone analogue on embryo survival after embryo transfer with both early- and late-stage embryos

After embryo transfer with both early- and late-stage embryos in goats, the reproductive outcomes of goat recipients are shown as Table 2. Transferring embryos at the late stage resulted in significantly higher pregnancy rates and significantly lower embryonic loss rates compared to transferring embryos at the early stage (p<0.05), regardless of the treatments. When early-stage embryos were transferred into recipients, pregnancy and kidding rates did not differ significantly between the control and GnRH analogue groups. However, the embryonic loss rate was significantly lower in the GnRH analogue group compared to the control (p<0.05). Conversely, when late-stage embryos were transferred, there were no significant differences in pregnancy rate, kidding rate, or embryonic loss rate between the GnRH analogue and control groups.

## DISCUSSION

According to various authors, the number of follicular waves per cycle ranges from two to five; however, for goats with a typical interovulatory cycle of 19 to 22 days, the predominant patterns are three and four waves [24–26]. Waves 1, 2, 3, and 4 (the ovulatory wave) appear on days 0, 5 to 6, 10 to 11, and around day 15 post-ovulation, respectively, whereas in goats with three follicular waves, wave 2 appears 1 to 2 days later and the ovulatory wave appears 1 to 2 days earlier [26]. To enhance the efficiency of estrous synchronization in this study, we compared two treatments: CI13D, based on data from goats with three follicular waves, and CI11D, based on data from goats with four follicular waves. Although the estrous synchronization efficiency did not differ significantly between treatments, the efficiency in terms of pregnancy rate, kidding rate, and number of kids was higher in the CI13D treatment compared to the CI11D treatment when different hormones were subsequently used to elevate progesterone levels and enhance luteal function. This was likely because most of the goats in this study had three follicular waves. When the CI13D, it aligned with the dominance phase of the second wave follicle, leading to higher quality eggs compared to the CI11D, where the second wave follicle was smaller. This assumption was supported by the plasma estradiol 17-β levels in the CI13D treatment, which showed a peak on D0AI and a rapid drop on D5AI, indicating ovulation occurred regardless of the hormonal groups. In contrast, the CI11D treatment showed no change in estradiol 17-β levels during the expected ovulation event. Our results suggest that the success of CIDR insertion depends on each animal's follicular wave count, indicating that tailoring protocols to these patterns can improve reproductive outcomes.

In experiment 1, although the differences in progesterone levels between the groups were not significant, it is interesting to note that the control group consistently exhibited lower progesterone levels across most time points compared to the groups treated with hCG and GnRH analogue. This subtle difference likely reflects the specific effects of the hormones used in the study. The hCG acts on LH receptors to promote luteal cell differentiation and accessory CL formation, potentially enhancing progesterone production through its luteotropic effects [27,28]. GnRH analogue, while primarily stimulating pituitary receptors to release LH and FSH, also exerts direct effects on the ovary, as evidenced by the presence of GnRH receptors in granulosa cells of preovulatory follicles and granulosa luteal cells [29]. A previous study has shown that induced CL by GnRH in cows develops into fully functional structures with normal weight and progesterone content comparable to spontaneously formed CL [30]. Despite these observations, administering GnRH analogue and hCG on day 5 post-AI did not result in significant differences in progesterone levels between the groups. This contrasts with previous studies, which found that applying a GnRH agonist on the day of estrus [31] or day 12 after mating increased post-treatment plasma P4 concentration [32,33], and using hCG on day 4 or day 7 post-AI significantly increased progesterone levels compared to the control [11,12]. This difference may be explained by variations in experimental conditions, such as the type, dose, or timing of GnRH analogue or hCG injection, as well as differences in the animals used, since some of the studies were conducted in ewes. Additionally, one limitation of this study is the frequency of blood sampling, which may not have been sufficient to capture transient changes in hormone levels following injections. This might explain the lack of significant differences in progesterone levels between groups. Increasing the sampling frequency in future studies could provide a more detailed hormone profile.

Though the use of GnRH analogue in this study did not significantly increase progesterone levels, it did result in significantly higher pregnancy rates, kidding rates, and the number of kids compared to the control group. Our results align with a previous study that found GnRH administration improved the reproductive performance of goats in terms of kidding rates when administered on day 12 post-mating. Administration of GnRH analogue leads to a rapid increase in plasma LH concentration and subsequently an increase in plasma progesterone levels in sheep [17,33]. It has been observed that the number of accessory CL increases with GnRH administration [17,33]. Although our study did not show a significant increase in plasma progesterone concentration nor did we determine the number of accessory CL, the effect of GnRH analogue on embryo survival in does could be attributed to the GnRH-stimulated LH surge. This surge may stimulate progesterone production by the CL and/or trigger ovulation, leading to the formation of accessory CL, which produce sufficient progesterone to support pregnancy, even if the increase was not statistically significant. Therefore, in experiment 2, we used GnRH analogue hormone to investigate whether it could enhance reproductive performance in does receiving transferred embryos.

When early-stage embryos were transferred into the oviduct, recipients given GnRH analogue hormone on day 5 after transfer had significantly lower embryonic loss rates compared to the control group. They also had higher pregnancy and kidding rates, although these increases were not statistically significant. Early embryonic mortality, a significant cause of pregnancy loss, is mainly attributed to luteal insufficiency [10,34]. The use of GnRH analogue supplements in this study has shown a positive effect, likely due to their role in reducing this early embryonic mortality. Consequently, GnRH analogue appear to support pregnancy and help maintain embryo survival until kidding. While the patterns of embryonic loss, pregnancy, and kidding rates showed no significant differences between the GnRH analogue and control groups for late-stage embryo transfers, significant differences were observed when comparing early-stage and late-stage transfers. Transferring late-stage embryos resulted in a significantly higher pregnancy rate and a significantly lower embryonic loss rate. This may be due to zygotic genome activation (ZGA), which mainly begins at the 4- to 8-cell stage in goats [35]. Developmental arrest is often observed during ZGA in embryos cultured *in vitro*, indicating that the onset of ZGA is crucial for early embryo development [36]. Studies have shown that blocking ZGA leads to improper gene expression and embryonic lethality [37,38]. Since the late-stage embryos used in this study were at the morula to blastocyst stages, they had already passed the critical ZGA phase. In contrast, the early-stage embryos were between the 2- to 8-cell stages, still early or in the midst of ZGA. This difference likely explains why transferring late-stage embryos resulted in better outcomes.

## CONCLUSION

In conclusion, this study demonstrates the significant impact of hormonal treatments on reproductive outcomes in goats. In Experiment 1, while the use of GnRH analogue did not significantly increase progesterone levels, it resulted in notably higher pregnancy rates, kidding rates, and the number of kids compared to the control group, demonstrating its positive effect on fertility. In Experiment 2, transferring late-stage embryos significantly improved pregnancy rates and reduced embryonic loss compared to early-stage transfers, likely because these embryos had already passed the critical phase of ZGA. Additionally, GnRH analogue supplementation supported pregnancy by reducing early embryonic mortality, which may be attributed to luteal insufficiency. Overall, although progesterone levels did not show significant differences, the use of a GnRH analogue enhanced reproductive outcomes, underscoring its potential to improve embryo survival and overall reproductive performance in goats.

## Figures and Tables

**Table 1:** Comparison of different hormone treatment groups was made based on pregnancy rate, kidding rate, number of kids, and average number of kids per litter between the CI11D and CI13D groups following artificial insemination with fresh semen

Hormone groups	Treatments		

	CI11D	CI13D	p-value
Control			
Pregnancy rate at 45 days	25.0 (2/8)	37.5 (3/8)^a^	1.000
Kidding rate	100.0 (2/2)	66.7 (2/3)^a^	1.000
Number of kids	3	3^a^	1.000
Number of kids per litter	1.5	1.5	
hCG			
Pregnancy rate at 45 days	25.0 (2/8)	62.5 (4/8)^ab^	0.606
Kidding rate	100.0 (2/2)	100.0 (4/4)^ab^	0.606
Number of kids	5	7^a^	0.334
Number of kids per litter	2.5	1.8	
GnRH analogue			
Pregnancy rate at 45 days	62.5 (5/8)	100.0 (8/8)^b^	0.200
Kidding rate	100.0 (5/5)	100.0 (8/8)^b^	0.200
Number of kids	13	23^b^	0.147
Number of kids per litter	2.6	2.9	
Progestogen			
Pregnancy rate at 45 days	37.5 (3/8)	62.5 (5/8)^ab^	0.617
Kidding rate	100.0 (3/3)	100.0 (5/5)^ab^	0.617
Number of kids	7	12^ab^	0.597
Number of kids per litter	2.3	2.4	

a,b Values with different superscripts in the same column are significantly different between groups and the same parameter (p<0.05).

CI11D, inserting the CIDR for 11 days; CI13D, inserting the CIDR for 11 days; hCG, human chorionic gonadotropin; GnRH, gonadotropin-releasing hormone; CIDR, controlled internal drug-releasing.

**Table 2:** Effect of GnRH analogue on pregnancy and kidding rates, litter size, number of transferrable embryos, and embryonic loss rate for early- and late-stage embryos

Groups	Pregnancy rate (%)	Kidding rate (%)	Number of kids	Number of kids per litter	Number of transferrable embryos	Embryonic loss rate (%)
Early-stage embryos	35.38 (23/65)	91.30 (21/23)	26	1.24	130	80.00 (104/130)
Control	27.59 (8/29)	87.50 (7/8)	7	1.00	58	87.93^a^ (51/58)
GnRH analogue	41.67 (15/36)	93.33 (14/15)	19	1.36	72	73.61^b^ (53/72)
Late-stage embryos	57.50 (23/40)	100.00 (23/23)	28	1.22	80	65.00 (52/80)
Control	50.00 (8/16)	100.00 (8/8)	9	1.13	32	71.88 (23/32)
GnRH analogue	62.50 (15/24)	100.00 (15/15)	19	1.27	48	60.42 (29/48)

a,b Values with different superscripts in the same column are significantly different between groups.

GnRH, gonadotropin-releasing hormone;.
